# sEMG-Based Neural Network Prediction Model Selection of Gesture Fatigue and Dataset Optimization

**DOI:** 10.1155/2020/8853314

**Published:** 2020-11-11

**Authors:** Fujun Ma, Fanghao Song, Yan Liu, Jiahui Niu

**Affiliations:** ^1^Center for Advanced Jet Engineering Technologies (CaJET), Key Laboratory of High-efficiency and Clean Mechanical Manufacture (Ministry of Education), National Experimental Teaching Demonstration Center for Mechanical Engineering (Shandong University), School of Mechanical Engineering, Shandong University, Jinan 250061, China; ^2^Department of Industrial Design, Mechanical Engineering, Shandong University, Jinan 250061, China

## Abstract

The fatigue energy consumption of independent gestures can be obtained by calculating the power spectrum of surface electromyography (sEMG) signals. The existing research studies focus on the fatigue of independent gestures, while the research studies on integrated gestures are few. However, the actual gesture operation mode is usually integrated by multiple independent gestures, so the fatigue degree of integrated gestures can be predicted by training neural network of independent gestures. Three natural gestures including browsing information, playing games, and typing are divided into nine independent gestures in this paper, and the predicted model is established and trained by calculating the energy consumption of independent gestures. The artificial neural networks (ANNs) including backpropagation (BP) neural network, recurrent neural network (RNN), and long short-term memory (LSTM) are used to predict the fatigue of gesture. The support vector machine (SVM) is used to assist verification. Mean square error (MSE), root mean square error (RMSE), and mean absolute error (MAE) are utilized to evaluate the optimal prediction model. Furthermore, the different datasets of the processed sEMG signal and its decomposed wavelet coefficients are trained, respectively, and the changes of error functions of them are compared. The experimental results show that LSTM model is more suitable for gesture fatigue prediction. The processed sEMG signals are appropriate for using as the training set the fatigue degree of one-handed gesture. It is better to use wavelet decomposition coefficients as datasets to predict the high-dimensional sEMG signals of two-handed gestures. The experimental results can be applied to predict the fatigue degree of complex human-machine interactive gestures, help to avoid unreasonable gestures, and improve the user's interactive experience.

## 1. Introduction

Unreasonable gesture is one of the reasons for the increase of hand lesions. To avoid injuries caused by improper gestures, the publishing paper titled Quantitative Analysis on the Interaction Fatigue of Natural Gestures selects three daily interactive gestures browsing information, playing games, and typing as the research objects and divides them into nine independent gestures. After denoising, filtering, segmenting, and extracting the parameters of the acquired surface electromyography (sEMG) signals, time-domain, frequency-domain, and time-frequency-domain characteristics are analysed. The characteristics of envelope waveform, power spectrum threshold, and fatigue of nine independent gestures are obtained. The long short-term memory (LSTM), one of the recurrent neural network (RNN) methods, is used to train nine independent gesture models. The fatigue characteristics of the integrated gestures are predicted by the trained LSTM series model. The energy consumption characteristics of integrated gestures in smartphones and PCs are obtained. It is found that the simple behaviour of browsing in the integrated behaviour is suitable for using natural interactive gestures, and the complex behaviours as playing games or typing of PC have lower energy consumption than that of smartphones. Among the independent natural gestures, the energy consumption of click is higher than that of dragging. Comparing the behaviours of the same purpose, the energy consumption of mouse gesture of PC is much lower than that of smartphone. The study of gesture fatigue provides a reference for the design of natural gesture and the development of Internet products. On this basis, this paper focuses on the selection of neural network prediction models and the optimization of datasets to improve the prediction accuracy of the model.

Artificial neural network (ANN) is also referred to as neural networks or connection models. It is an algorithmic mathematical model that imitates the behaviour characteristics of animal neural networks and carries out distributed parallel information processing [1]. This kind of network depends on the complexity of the system and achieves the purpose of processing information by adjusting the relationship between a large number of internal nodes. Moh [2] used the ANN classifier to test the two different feature sets including all principal components and selected principal components. Joga [3] used the trained neural network to embed into the wearable extra robotic fingers to control the robotic motion and assist the human fingers in bimanual object manipulation tasks. With the improvement of the algorithm, neural network models have been widely applied in many industries.

There is a very extensive research foundation to establish a variety of evaluation methods of prediction model such as ANN and support vector machine (SVM). Li [4] reported on the evaluations of five different machine learning algorithms, ANN, support vector regression (SVR), least-square support vector machine (LS-SVM), Gaussian process regression (GPR), and Gaussian mixture model (GMM), applied to four residential datasets that contain smart meters. Vega [5] evaluated and compared two common methods, ANN and SVR, for predicting energy productions from a solar photovoltaic system in different times. Zheng [6] proposed a novel approach combing wavelet technique with LS-SVM for forecasting of dissolved gases in oil-immersed power transformers, and the mean absolute percentage errors of the proposed approach are significantly better than that of BPNN, radial basis function neural network, generalized regression neural network, and SVM regression (SVR). Hima [7] studied the applicability of LS-SVM for estimating the blast-induced flyrock. For comparison aim, SVR was also employed. Six machine learning models, including ANN, SVR, classification and regression tree, bagging regression tree, least absolute shrinkage and selection operator, and GPR, were applied to predict the bending force in the hot strip rolling process [8]. Nourani et al. [9] employed several artificial intelligence-based techniques including SVR, adaptive neurofuzzy inference system, ANN, and multiple linear regression models for ahead predictions of climatic stations in Iraq. Hassan [10] built a hybrid framework consisting of eleven SVRs implemented in Proteus 6/MATLAB environments. Data collected over seven years in a city of the north of Spain were analysed by Nieto using four different mathematical models: vector autoregressive moving-average, autoregressive integrated moving-average, multilayer perceptron neural networks, and SVMs with regression [11].

To further improve the prediction accuracy of neural networks, many scholars tried to adjust the super parameters of the neural networks, optimize the data models [12–14] and the algorithms [15], and use the hybrid neural network [16] and other methods. Chen [17] used the combination of neural networks of convolutional neural network (CNN) and LSTM to predict multi-DoF finger force and obtained the best performance. Arvind [18] introduced a transfer-learning-based Long-term Recurrent Convolution Network named as “MyoNet” for the classification of lower limb movements, along with the prediction of the corresponding knee joint angle. Arjunan [19] proposed a model employing the deep architecture combining CNNs and RNNs to estimate EMG-based limb movement. However, these methods are only applicable to specific research objectives and are not universal. This paper tries to optimize the dataset to further improve the prediction accuracy.

## 2. Previous Works

### 2.1. Prediction Models

There are many kinds of neural networks with different advantages and characteristics. The neural network models used for prediction include BP neural network, RNN, and LATM model. These prediction neural networks have advantages and disadvantages during testing. According to the characteristics of neural networks, many scholars established matching neural network models to solve different problems.

BP neural network was proposed by Rumelhart and McClelland in 1986. It is a multilayer feedforward network composed of nonlinear transformation units and trained according to error backpropagation algorithm. Li [20] utilized the BP neural network to recognize the imagery tasks. Chen et al. [21] used a BP neural network to map the optimal surface EMG features to the flexion/extension joint angles. Lei [22] used the neural network toolbox of MATLAB to train BP neural network and tested the established continuous movement control model. The advantage of BP neural network is that it has strong nonlinear mapping ability and flexible network structure. However, there are also some defects, such as slow learning speed, easy to fall into local minimum value, lack of theoretical guidance in the selection of the number of network layers and neurons. BP neural network is mainly including input layer, hidden layer, and output layer. Each layer is composed of several neurons. The neurons between the layers are fully connected, and the information flows from the input to the output in one direction. BP neural network adopts the learning rules of the gradient descent method. The core of the algorithm is to backpropagate the error between the target output of the input layer and the calculation value of the output layer from output to input layer by layer, and assign to each connection point. The calculated reference errors of connection points are used to adjust the thresholds and weights of the network, which benefits the expected output of the network approximating to the actual output.

RNN is a kind of neural network which takes sequence data as input, recursion in the evolution direction of sequence, and all nodes (cyclic units) are linked in chain. RNN has characteristics of memory, parameter sharing, and Turing completion, so it has certain advantages in learning nonlinear features of sequences. RNN has been applied in natural language processing, time series prediction, and so on. Huang et al. [23] used deep-recurrent neural networks to predict the real-time intended knee joint motion. Li et al. [24] embedded Kalman filter and RNN into the real-time functional electrical stimulation system for identification and estimation. Kudithipudi [25] proposed a neuromemristive reservoir computing architecture with doubly twisted toroidal structure that significantly improved RNN architecture with an accuracy of 90 and 84% for epileptic seizure detection and EMG prosthetic finger control, respectively. However, the challenge in using RNN is the very unstable relationship between parameters and the dynamics of hidden states, known as “fading or exploding gradients.” Therefore, LSTM and gate recurrent unit gating systems are proposed. The typical structure of RNN is consistent with that of neural network, which is composed of input layer, hidden layer, and output layer. Different from the input and output of the fixed dimension feedforward neural network, the neurons in RNN have self-feedback and cyclic structure. The inputs of interconnected neurons in the hidden layer include not only the weight and input of the current time *t*, but also the output of the hidden unit *t* − 1 at the previous time and all the previous moments.

LSTM is an important branch of RNN. It solves the vanishing gradient problem caused by the gradual reduction of the gradient backpropagation process. LSTM model can store the input memory of long-time steps when processing time series information, and the insertion of random time steps on the input sequence has robustness. Dao [26] developed and evaluated a LSTM model as a recurrent deep neural network to transfer learning for the prediction of skeletal muscle forces. Chen et al. [27] used LSTM to verify the improvement of the estimated accuracy of the continuous estimation model of upper limb joint angles proposed by them. LSTM can solve the problem of RNN gradient to a certain extent, but not completely. Because the structure of each cell has a full connection layer, LSTM has drawbacks of large calculation, low efficiency, and slow speed.

SVM is a kind of generalized linear classifier which classifies data by supervised learning. Its decision boundary is the maximum margin hyperplane to solve the learning samples [28]. SVM was proposed in 1964 and has been developed rapidly since the 1990s. A series of improved and extended algorithms have been developed and applied in pattern recognition. Cao [29] compared the performance of the CNN model, the model based on SVR, and the model based on partial least-square regression. Barenya [30] compared the prediction model of COVID-19 with the state-of-the-art SVR model and the conventional RVFL model.

Pontes [31] proposed a novel flexible hierarchical age estimation approach consisting of a multiclass SVM to classify a subject into an age group followed by an SVR to estimate a specific age. Koerich [32] compared rainfall-runoff modelling between an SVM-based approach and the EPA's storm water management model. SVR shows great potential for applications in the field of urban hydrology, but the algorithm tends to underestimate the peak discharge, which has significant limitations regarding the model calibration. Kang [33] proposed a semisupervised SVR method based on self-training.

The core idea of SVM is to construct a hyperplane in an *n*-dimensional space to distinguish different classifications in the feature space. To calculate the distance between the two types of patterns, the algorithm constructs two hyperplanes parallel to the classification surface on both sides of the classification surface, and the data on these two hyperplanes are called support vectors. For linear inseparable data, the classification idea of SVM is to map the inseparable data into a high-dimensional space through nonlinear transformation, so that the original unclassifiable data can be transformed into high-dimensional separable data. At the same time, the data are divided by solving the optimal classification hyperplane in the high-dimensional space to achieve the effect of data classification [34]. SVM has the advantages of good generality and robustness, simple, effective, and perfect theory. Therefore, SVM has many unique strengths in solving the problems with small sample, nonlinear, and high-dimensional pattern recognition.

BP neural network, RNN, and LSTM are widely used in the prediction of gesture fatigue. The SVR algorithm of SVM is used in the predictive model. However, SVM is inefficient for large-scale training samples, and it is difficult to solve the multiclassification problem. In this paper, more than 10000 sets of sEMG signals are collected by integrated gestures, so SVR is only used as the reference of neural network model in a small sample fatigue prediction model of independent gestures.

### 2.2. Prediction Model Datasets

Many scholars attempt to optimize datasets to further improve the accuracy of the neural network prediction model. Zhao [35] used the mean absolute value (MAV), variance (VAR) of time-domain features, median frequency (MF), average power frequency (MP) of frequency-domain features, and wavelet decomposition coefficients to recognize gesture by BP neural network. It was found that the recognition rate of the fusion features was significantly higher than that of the single feature. It is more efficient to use VAR + MMV (modulus maximal of wavelet transform coefficients) as the feature for pattern recognition of sEMG signals. Wu et al. [36] obtained the optimal measurement position sets for gesture recognition when different feature sets were used, and similarly, the optimal feature sets when different measurement position sets were used. The results showed that the error function can be effectively reduced by using appropriate datasets for different problems. Therefore, this paper uses different sEMG signal features to test and find suitable datasets for gesture fatigue prediction.

## 3. Materials and Methods

### 3.1. Experimental Process and Data Processing

**Figure 1:**
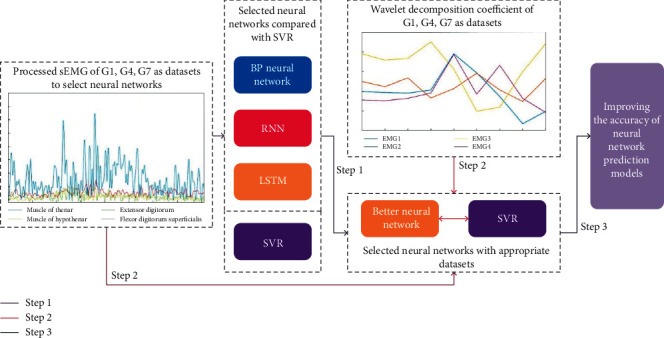
Optimization flowchart of the prediction model.

First, to evaluate the accuracy of the prediction, BP neural network, RNN, and LSTM methods are used to train the sEMG signals of independent gestures in this paper, and SVR is used as the reference. The more effective neural network is selected according to the MSE, RMSE, and MAE values of evaluation indexes of prediction. Second, after the type of neural network is determined, the influence of different datasets (the processed sEMG signals and wavelet decomposition coefficients) on the prediction accuracy of the model is explored. Finally, combine the optimizations of Steps 1 and 2 to improve the accuracy of neural network prediction models, as shown in Figure 1.

**Figure 2:**
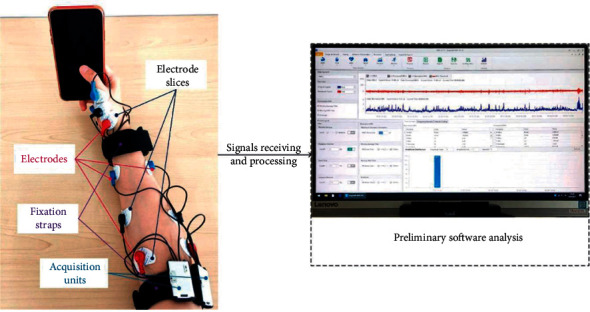
Illustration of sEMG signal acquisition system.

EMG wireless signal collector and Ergolab software of Beijing Jinfa Technology Co., Ltd., are used to collect original sEMG signals. There are 5 postgraduates aged 20–35 years as subjects. The width and thickness of their hands are in accordance with the national standard GB/T 26158. The electrode slices, electrodes, acquisition unites, and fixation straps are used for sEMG signal acquisition of independent and integrated gestures. The sEMG signals have been received and preliminary processed to the computer, as shown in Figure 2. The acquisition frequency of the original sEMG signal is 1024 Hz.

**Figure 3:**
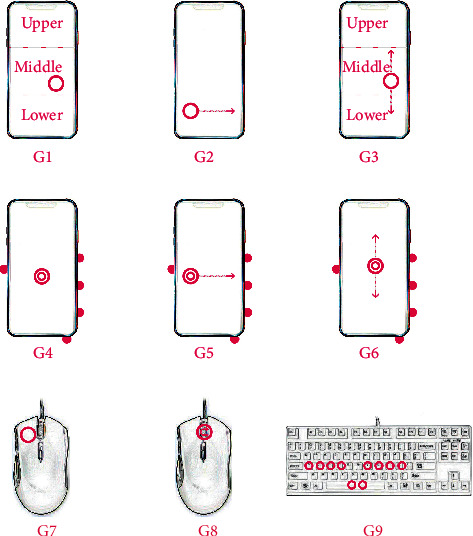
Illustrations of G1-G9 independent gestures.

In Figure 3, the circles represent the click and the arrows indicate the sliding direction. The turning arrow of G8 indicates rolling. The solid dots on the outside of the smartphones in G4-G6 indicate the second supporting. G1, G2, and G3 are one-handed click gesture, horizontal drag, and vertical drag, respectively. G4, G5, and G6 are two-handed click, horizontal drag, and vertical drag, respectively. G7 is one-handed mouse click gesture. G8 is the middle mouse button scroll gesture. G9 is two-handed keyboard input gesture.

In this paper, three comprehensive gestures of browsing information, playing games, and typing are divided into nine independent gestures, as shown in Figure 3. To explore the influence of large-screen smartphones on fatigue in more detail, the screens are divided into upper, middle, and lower parts in G1 and G3 gestures. The circles represent the click and the arrows indicate the sliding directions. The turning arrow of G8 indicates rolling. The solid dots on the outside of the smartphones in G4-G6 indicate the second supporting. Due to the large difference of the nine independent gestures, these gestures can be classified into three categories: one-handed gesture based on smartphone, two-handed gesture based on smartphone, and gesture based on PC. For each category, one-handed G1-lower gesture, two-handed G4 gesture, and G7 with mouse click are selected as feature sets of neural network prediction. According to the principle of anatomy, the sEMG signals of thenar and hypothenar muscles, extensor digitorum, and flexor digitorum superficialis are measured as four channels of one-handed gesture. The two-handed gesture of 7 channels also includes the sEMG signals of the thenar and hypothenar muscles and the extensor digitorum of the left hand.

In this paper, the original sEMG signals are filtered by Chebyshev third-order low-pass filter. The band pass and band stop of the filter are 55 Hz and 90 Hz, respectively, and normalized. A group of periodic signal length as window length is selected by each gesture to cut signals. Due to different data characteristics, the window length is selected according to the actual signal characteristics, for example, the window length of G1 is 15 and the window length of G4 and G7 is 20. The processed sEMG signals are used as the feature datasets of the training models. The power spectrums of the processed sEMG signals are extracted in the frequency domain and used as the label dataset of the processed sEMG signals. The parameters of wavelet decomposition in the time-frequency domain are extracted as the backup feature dataset.

To speed up the learning efficiency of neural network prediction models, the input and output datasets of processed sEMG signals are standardized as follows:

(1) $$\begin{array}{c} x_{k}^{\text{new}}=\frac{x_{k}^{\text{old}}-\min x_{k}^{\text{old}}}{\max x_{k}^{\text{old}}-\min x_{k}^{\text{old}}}, \end{array}$$

(2) $$\begin{array}{c} y_{k}^{\text{new}}=\frac{y_{k}^{\text{old}}-\min y_{k}^{\text{old}}}{\max y_{k}^{\text{old}}-\min y_{k}^{\text{old}}}, \end{array}$$

where *k* represents the number of samples, *x*_*k*_^old^ and *y*_*k*_^old^ are input and output datasets before network processing, and *x*_*k*_^new^ and *y*_*k*_^new^ are input and output datasets after network processing. In this paper, the sEMG signals are normalized to the threshold of (0, 1). According to the formula principle of SVM, the decision boundary is divided according to (−1, 1), and the normalized threshold of (−1, 1) is also used in the validation of SVR model.

### 3.2. Prediction Model Training

According to literature research and the characteristics of sEMG signals, such as multidimensional, time series, and large amount of data, BP neural network, RNN, and LSTM are chosen as training models to predict fatigue in this paper.

**Figure 4:**
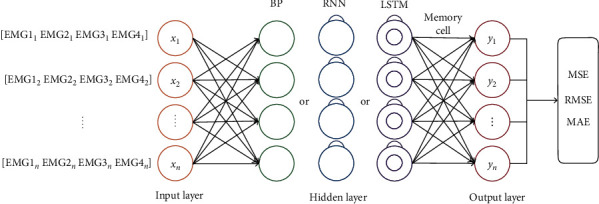
BP, RNN, and LSTM neural network prediction model training.

The input matrixes of one-handed gestures are 4-channel signals, while those of two-handed gestures are 7-channel signals. To compare the training results, the input sequence is set as a matrix of 4 vectors with 4 dimensions for each vector. The epochs of models are 200 times, and 120 groups of data are trained in each batch. To optimize the prediction model, regularizers of l1 and l2, optimizers of Adam, and stochastic gradient descent (SGD) are widely used in this paper. After many times of training, compared with the optimal gradient descent, the super parameters and optimizer of each prediction model are different. The hidden layer neurons of BP neural network are independent neurons, hidden layer neurons of RNN have continuous time series characteristics, and hidden layer neurons of LSTM are memory neurons, as shown in Figure 4. The error values of MSE, RMSE, and MAE are calculated between the output layer and the original *y* value.

#### 3.2.1. BP Neural Network

A three-layer BP neural network structure with one hidden layer is used in this paper. The sigmoid activation function is used from input layer to hidden layer. The optimizer uses Adam with a learning rate of 10^−4^.

Let the dataset of input layer be *x*, the weight and bias from input layer to hidden layer be *w* and *b*_1_, and that from hidden layer to output layer be *v* and *b*_2_. Then the equation from input layer to hidden layer is as equation (3). The equation from the hidden layer to the output layer is as equation (4):

(3) $$\begin{array}{c} {\text{net}}_{\text{input}-\text{hidden}}=w^{T}x+b_{1}, \end{array}$$

(4) $$\begin{array}{c} {\text{net}}_{\text{hidden}-\text{output}}=v^{T}x+b_{2}. \end{array}$$

(1) Import feature and label datasets. The weights and biases in the initialization network are recorded as *w*^(0)^, *b*_1_^(0)^, *v*^(0)^, *b*_1_^(0)^, respectively.
(2) Activate forward propagation, and obtain the expected values of the output and loss functions of each layer:
  (5) $$\begin{array}{c} E\left(\theta \right)=\frac{1}{n}\overset{n}{\underset{i=1}{\sum }}{\left(y_{i}-{\hat{y}}_{i}\right)}^{2}, \end{array}$$

where $\theta ,y_{i},{\hat{y}}_{i}$ represent the parameter set, real value, and predicted value, respectively, and *n* represents the output of *n*-dimensional data.
(3) Calculate the error terms of output unit and hidden unit according to the loss function.
Calculating the error term of the output unit is to calculate the gradient value or partial derivative of the loss function with respect to the output unit as follows:
  (6) $$\begin{array}{c} \nabla_{\left(k\right)}v=\frac{\partial E}{\partial v}, \end{array}$$
  (7) $$\begin{array}{c} \nabla_{\left(k\right)}b_{2}=\frac{\partial E}{\partial b_{2}}. \end{array}$$

  The error term of hidden unit is calculated as follows:

(8) $$\begin{array}{c} \nabla_{\left(k\right)}w=\frac{\partial E}{\partial w}, \end{array}$$

(9) $$\begin{array}{c} \nabla_{\left(k\right)}b_{1}=\frac{\partial E}{\partial b_{1}}. \end{array}$$

(4) Update weights and bias terms in BP neural networks.
  Update the parameters of output unit as follows:
    (10) $$\begin{array}{c} v^{\left(k\right)}=v^{\left(k-1\right)}-\eta \nabla_{\left(k\right)}v, \end{array}$$
    (11) $$\begin{array}{c} b_{2}^{\left(k\right)}=b_{2}^{\left(k-1\right)}-\nabla_{\left(k\right)}b_{2}. \end{array}$$

Update the parameters of hidden unit as follows:
  (12) $$\begin{array}{c} w^{\left(k\right)}=w^{\left(k-1\right)}-\eta \nabla_{\left(k\right)}w, \end{array}$$
  (13) $$\begin{array}{c} b_{1}^{\left(k\right)}=b_{1}^{\left(k-1\right)}-\eta \nabla_{\left(k\right)}b_{1}, \end{array}$$

  where *η* represents the learning rate and *k* = 1, 2, ..., *n* represents the number of updates or iterations.
(5) Repeat steps 2–4 until the loss function is less than the given threshold or the number of iterations is reached. The output parameters at this time are the current optimal parameters.

#### 3.2.2. RNN

In this paper, a three-layer RNN structure is adopted. The l2 regularizer is used in each layer of regularization. The activation function of output layer is sigmoid. The SGD optimizer is used.

**Figure 5:**
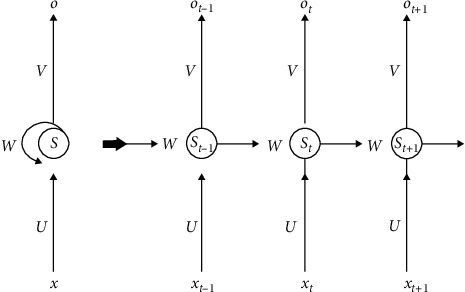
Schematic diagram of RNN structure.

The output structure of RNN is shown in Figure 5. The parameters *t*, x, *s*, and *o* are the basic parameters of network structure, which represent time sequence, input layer, hidden layer, and output layer, respectively. The parameters *U* and *W* are the offset and weight of transfer function.

The input time series of sEMG signals are *x* = (*x*^(1)^,…, *x*^(*T*)^), and the basic RNN calculates the hidden element sequence **h** = (**h**^(1)^,…, **h**^(*T*)^) and the output sequence of power spectrum **o** = (**o**^(1)^,…, **o**^(*T*)^) by iterating the following equations:

(14) $$\begin{array}{c} h^{\left(t\right)}=f\left(W_{xh}x^{\left(t\right)}+W_{hh}h^{\left(t-1\right)}+b_{h}\right), \end{array}$$

(15) $$\begin{array}{c} o^{\left(t\right)}=g\left(W_{ho}h^{\left(t\right)}+b_{o}\right), \end{array}$$

where *t* = 1,…, *T*, *W* represents the weight matrix (e.g., *W*_*xh*_ is the weight matrix of input to the hidden element), and *b* is the offset vector (e.g., *b*_*h*_ is the offset vector of the hidden layer), and the activation function of the hidden layer is represented by *f* and *g*.

The parameters in the model are shared at each time, so that the model can be easily extended to samples of different lengths and generalized. Moreover, parameter sharing prevents the overall parameters of the network from increasing with the increase of data volume, which is convenient for training. The cost function is defined as follows:

(16) $$\begin{array}{c} L_{1}=\frac{-1}{NT}\overset{N}{\underset{n=1}{\sum }}\overset{T}{\underset{t=1}{\sum }}\left(\overset{K}{\underset{i=1}{\sum }}y_{\left(ni\right)}^{\left(t\right)}\log\left(o_{\left(ni\right)}^{\left(t\right)}\right)\right), \end{array}$$

where *N* represents the sample number of input data, *T*  is the total number of times, *K* is the dimension of model output at each time, *o*(*t*) is the output of the *t*_th_ moment, and *y*(*t*) is the target output at the *t*_th_ moment.

The parameters of RNN can be trained by the backpropagation through time algorithm [37], that is, from the end of the sequence, it is calculated reversely. For each node in the model, the gradient of the node needs to be calculated recursively based on the gradient of the next node.

#### 3.2.3. LSTM

A LSTM predictive model with a hidden layer is used. To prevent overfitting, the dropout of hidden layer uses the value of 0.1. When the optimizer is Adam and the learning rate is 10^−4^, the convergence effect of loss function is the best.

**Figure 6:**
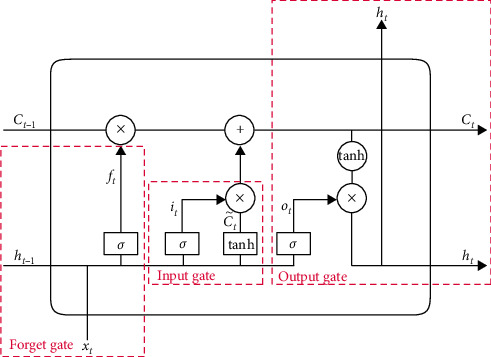
Diagram of LSTM structure.

Three important gate control functions are introduced into the memory cell module of the hidden layer in LSTM. The three gate control functions realize three kinds of gate control functions: forgetting gate, input gate, and output gate. In the forward propagation process of LSTM, the storage and interaction of information are controlled by the three gate structures of the hidden layer memory unit, as shown in Figure 6.

(1) Forget gate *f*_*t*_ is used to control the proportion of time span of input information and determine how much of the cell state *C*_*t-*1_ of last moment is left to current moment. The proportional control is realized by the parameter *σ* whose value range is (0, 1).
  The sigmoid function is used to control the superposition of hidden layer *h*_*t*−1_, output layer, and input layer *x*_*t*_. The output expression *f*_*t*_ of forget gate is as follows:

(17) $$\begin{array}{c} f_{t}=\sigma \left(W_{f}\cdot \left[h_{t-1},x_{t}\right]+b_{f}\right). \end{array}$$

(2) The input gate *i*_*t*_ controls the input process of information in the current time and determines how much of the input *x*_*t*_ of the network at the current time is saved to cell state *C*_*t*_. This process includes the update process of the information in the current time completed by the input gate and superimposes the input of the previous time on the hidden layer to the current state to realize the dependence on the time sequence information. The input gate function includes a sigmoid function and a tanh function. The calculation method is as follows:

(18) $$\begin{array}{c} i_{t}=\sigma \left(W_{i}\cdot \left[h_{t-1},x_{t}\right]+b_{i}\right). \end{array}$$

  Candidate $\tilde{C_{t}}$ is used to conclude new knowledge to be stored in cell state. The calculation method for the update process of the information in current time is as follows:

(19) $$\begin{array}{c} \bar{C_{t}}=\tanh\left(W^{C}x_{t}+U^{C}h_{t-1}\right). \end{array}$$

  The superposition process of the output of the previous time on the hidden layer and the current input is shown in the following equation:

(20) $$\begin{array}{c} C_{t}=f_{t}*C_{t-1}+i_{t}*\bar{C_{t}}. \end{array}$$

  At the current moment *t*, according to the combined action of the forget gate and input gate, the memory unit of hidden layer completes the information multiplication operation and realizes the output of the memory unit cell state *C*_*t*_.
(3) The output gate *O*_*t*_ controls how much of unit status *C*_*t*_ outputs to the current output value *h*_*t*_ of LSTM. Before exporting the memory unit information, the output gate function controls the output information and returns the timing information to the hidden layer. This process updates the information state of the memory unit. The calculation method of output gate is shown in the following equation:

(21) $$\begin{array}{c} O_{t}=\sigma \left(W_{O}\cdot \left[h_{t-1},x_{t}\right]+b_{o}\right). \end{array}$$

The information of memory state *h*_*t*_ returned to hidden layer is calculated as follows:

(22) $$\begin{array}{c} h_{t}=O*\tanh\left(C_{t}\right). \end{array}$$

#### 3.2.4. SVM

This paper uses an SVR prediction model. When the penalty parameter *C* is 10, kernel type is RBF function, and gamma uses “auto,” the prediction result is stable, and error function is low.

The sEMG training dataset of SVM is T = {(*x*_1_, *y*_1_), (*x*_2_, *y*_2_)}, where *x*_*i*_ is the eigenvector used for classification, *y*_*i*_ is the category label, and *y*_*i*_ ∈ {1, −1}, and SVM optimizes data segmentation by constructing the following hyperplane:

(23) $$\begin{array}{c} \left(\omega \cdot x\right)+b=0. \end{array}$$

The above equation needs to satisfy the conditions as follows:

(24) $$\begin{array}{c} \left\{\begin{array}{cc} \left(\omega \cdot x_{i}\right)+b\geq 1, & y_{i}=1,\\ \left(\omega \cdot x_{i}\right)+b\leq 1, & y_{i}=-1, \end{array}\right. \end{array}$$

(25) $$\begin{array}{c} y_{i}\left(\omega \cdot x_{i}\right)+b\geq 1, \end{array}$$

where *i* ∈ {1,…, *N*}. The optimal classification plane needs to satisfy the condition that the distance between the two support planes is the largest:

(26) $$\begin{array}{c} d=\frac{2}{\left\|\omega \right\|}. \end{array}$$

To find max(2/‖*ω*‖), it can be transformed into the following equation:

(27) $$\begin{array}{c} \min\frac{{\left\|\omega \right\|}^{2}}{2}, \end{array}$$

where *i* ∈ {1,…, *N*}, and *s*.*t*., *y*_*i*_(*ω* · *x*_*i*_) + *b* ≥ 1. The original problem can be transformed into a dual optimization problem by introducing Lagrange multiplier *α*. The original equation can be changed as follows:

(28) $$\begin{array}{c} ℒ\left(\omega ,b,\alpha \right)=\frac{\left\|\omega^{2}\right\|}{2}-\overset{n}{\underset{i=1}{\sum }}\alpha_{i}\left(y_{i}\left(\omega x_{i}+b\right)-1\right). \end{array}$$

Through Lagrange multiplier, solving the problem is equivalent to finding the maximum value of *L* function. Through the process of solving the dual problem, the *L* function can be expressed as follows:

(29) $$\begin{array}{c} ℒ\left(\omega ,b,\alpha \right)=\overset{n}{\underset{i=1}{\sum }}\alpha_{i}-\frac{1}{2}\overset{n}{\underset{i,j=1}{\sum }}a_{i}a_{j}y_{i}y_{j}x_{i}^{T}x_{j}, \end{array}$$

where the constraint condition is *α*_*i*_ ≥ 0, and ∑_*i*=1_^*n*^*a*_*i*_*y*_*i*_ = 0. The question of solving equation (27) can be transformed into finding the maximum value of ℒ(*ω*, *b*, *α*). The final problem is to find the appropriate *a*_*i*_ making ℒ maximum. Supposing obtained *a*_*i*_ is recorded as *a*_*i*_^*∗*^, the normal vector *ω*^∗^ and intercept *b*^∗^ of the corresponding plane can be expressed as follows:

(30) $$\begin{array}{c} \omega^{*}=\overset{n}{\underset{i=1}{\sum }}\alpha_{i}^{*}x_{i}y_{i}, \end{array}$$

(31) $$\begin{array}{c} b^{*}=y_{sv}-\overset{n}{\underset{i=1}{\sum }}\alpha_{i}^{*}y_{i}<x_{i},x_{sv}>, \end{array}$$

where *x*_*sv*_ and *y*_*sv*_ are the corresponding data of support vector. 〈*x*_*i*_, *x*_*sv*_〉 is the inner product of *x*_*i*_ and *x*_*sv*_.

For the nonlinear separable data of sEMG signals, the kernel function is used to map data from low-dimensional space to high latitude. Using the parameter *K* to represent the kernel function, the question can be expressed as follows:

(32) $$\begin{array}{c} f\left(x\right)=\underset{i\in SV}{\sum }\alpha_{i}^{*}y_{i}K\left(x_{i},x_{sv}\right)+b^{*}. \end{array}$$

The commonly used kernel functions include linear kernel, polynomial kernel, radial basis function (RBF), and sigmoid tank [38]. Gaussian function is used in this paper. The expression of Gaussian function is as followsfd33:

(33) $$\begin{array}{c} K\left(x_{1},x_{2}\right)=\exp\left(\frac{\left\|x_{1}-x_{2}^{2}\right\|}{2\delta^{2}}\right), \end{array}$$

where *x*_1_ and *x*_2_ are two points in the sample space, and *x*_1_, *x*_2_ ∈ *R*^*n*^. *δ* is the kernel radius and *δ* > 0.

### 3.3. Dataset Optimization

#### 3.3.1. Wavelet Transform Theory

Wavelet transform (WT) theory is a signal analysis tool. By using the methods of dilation and translation, the displacement *r* of the base wavelet function *ψ*(**t**) is calculated, and different scales of *a* does the inner product with the signal *f*(*t*) in WT theory:

(34) $$\begin{array}{c} {\text{WT}}_{f}\left(\alpha ,\tau \right)=\frac{1}{\sqrt{a}}\int_{-\infty }^{+\infty }f\left(t\right)\psi^{*}\left(\frac{t-\tau }{\alpha }\right)\text{d}t,a>0. \end{array}$$

The frequency-domain equivalent expression of WT is as follows:

(35) $$\begin{array}{c} {\text{WT}}_{f}\left(\alpha ,\tau \right)=\frac{\sqrt{\alpha }}{2\pi }\int F\left(\omega \right)\Psi^{*}\left(\alpha \omega \right)e^{j\omega t}\text{d}\omega ,a>0, \end{array}$$

where *F*(*ω*), Ψ^*∗*^(*αω*) are the Fourier transforms of *f*(*t*) and *ψ*(**t**), respectively.

According to the structure chart of wavelet decomposition tree, the multiresolution characteristic of wavelet decomposition is only reflected in the low-frequency part. The decomposition process can carry out secondary decomposition of low-frequency components, until the high-frequency components are obtained. Therefore, the sEMG signal *S* is decomposed into a high-frequency component *d*_1_ and a low-frequency component *a*_1_, as shown on the left side of Figure 4. The low-frequency component *a*_1_ is decomposed into a low-frequency component *a*_2_ and a high-frequency component *d*_2_ again. The final formula is as follows:

(36) $$\begin{array}{c} S=a_{2}+d_{2}+d_{1}. \end{array}$$

#### 3.3.2. Wavelet Decomposition Coefficient

**Figure 7:**
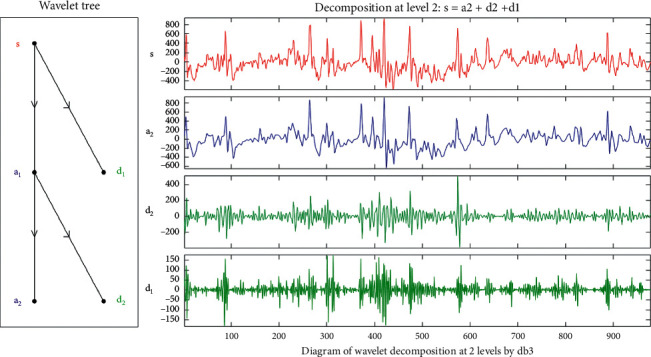
The wavelet decomposition diagram of G1 gesture of sEMG signals in channel 1.

Wavelet decomposition is widely used in the research field of gesture recognition, and it can obtain low-frequency and high-frequency decomposition coefficients of sEMG signals, which are more significant than the characteristics of the original signals. In this paper, Daubechies 3-order basis wavelet is used to decompose the processed sEMG signals in two layers. Two relative energy coefficients representing different frequency components are extracted to form a 20-dimensional feature samples. As shown in Figure 7, the left diagram is the wavelet decomposition tree of sEMG signal channel 1 of gesture G1, and the right figure is the waveform diagram of detailed wavelet decomposed signals.

## 4. Results and Discussion

There are many evaluation criteria for prediction models. In this paper, MSE, RMSE, and MAE are used as the calculation functions of accuracy. MSE is the expected value of the squares of the differences between the estimated values and the true values of the parameter. MSE can be used to evaluate the degree of change in data. The smaller the MSE value is, the better the prediction model is to describe the experimental data:

(37) $$\begin{array}{c} \text{MSE}=\frac{1}{n}\sum_{i=1}^{n}{\left(y_{\text{real}}-y_{\text{predict}}\right)}^{2}. \end{array}$$

RMSE is an evaluation criterion to measure the error rate of the regression model. When the predicted value and the real value are completely consistent in the range of [0, +∞), it is equal to 0, that is, the perfect model. The greater the error, the greater the value:

(38) $$\begin{array}{c} \text{RMSE}=\sqrt{\frac{1}{n}\sum_{i=1}^{n}{\left(y_{\text{real}}-y_{\text{predict}}\right)}^{2}}. \end{array}$$

MAE is the mean absolute error of given data points, in the same units as the original data. Generally, the smaller the value, the better the fitting effect of the model:

(39) $$\begin{array}{c} \text{MAE}=\frac{1}{n}\sum_{i=0}^{n-1}\left|y_{\text{real}}-y_{\text{predict}}\right|. \end{array}$$

RMSE and MAE can only compare models' error with the same unit. The magnitude of MAE is approximated to that of RMSE, but its error value is relatively small.

### 4.1. Prediction Model Selection

The different neural network test results of three groups of sEMG signals after treatment are shown in Table 1. Each group is trained with 200 epochs, and each epoch has a batch size of 120. Each group of sEMG signals is divided into training set and test set for evaluation. Since SVR uses Gaussian regression kernel to calculate the problems and has a clear training process, the predicted evaluation value has good robustness. The MSE values of BP neural network, RNN, and LSTM are fixed, while the evaluation values of RMSE and MAE are different with the random gradient decrease. However, the difference of that is small. The optimal values of random 5 times are obtained and compared.

The comparison results show that the prediction effect of BP neural network is poor. The MSE value of three groups of sEMG signals is ideal, but the RMSE of G1 training set and test set is as high as 0.34, and MAE is more than 0.3. The training effect of SVR is good. MSE, RMSE, and MAE of SVR are all in a low level. Both RNN and LSTM are neural network models suitable for time series characteristics. The results of prediction training show that the three groups of signals of the two models perform the same in MSE which cannot be compared. In RMSE evaluation, the results of LSTM in G1 and G7 groups are better than that of RNN, but that of G4 group is slightly worse than RNN. It shows that the training LSTM model has more advantages in shorter dimension datasets.

**Figure 8:**
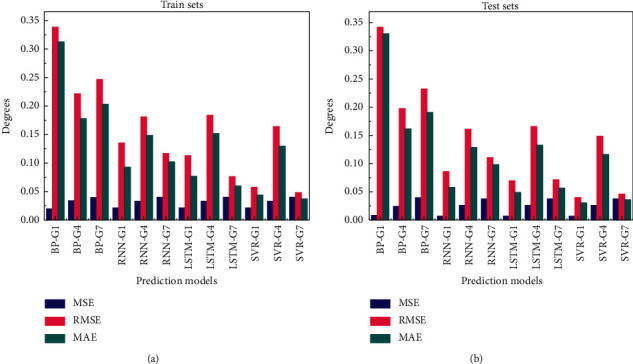
Comparison chart of MSE, RMSE, and MAE for sEMG-based prediction models.

For more intuitive comparison, the results of three groups of prediction models based on sEMG signal of each neural network are shown in Figure 8. Blue represents MSE value of each group of models, and pink and green represent RMSE and MAE values, respectively. The prediction evaluation result of the training sets is shown in Figure 8(a), and that of the test sets in Figure 8(b).

It can be seen intuitively that the error value of BP neural network in the prediction effect of three group gestures is significantly bigger than that of other models. Therefore, the BP model is excluded and will not be discussed any more. The average prediction errors of RNN, LSTM, and SVR are similar. The values of evaluation index of two-hand G4 gesture are higher than that of one-hand gesture. SVR has the lowest average error in small samples G1, G4, and G7, and it is one of the suitable predictive models to study gesture fatigue.

Since the sEMG signals of hand gestures belong to time series datasets, RNN and LSTM models are more suitable for the prediction of gesture movement trend. According to the comparison in Table 2 and Figure 8, the prediction error of LSTM in G1 and G7 is slightly lower than that of RNN, and that in G4 is slightly higher than that of RNN. Therefore, according to the comparison results, SVR and LSTM as superior models are selected in this paper for optimization training in the next step.

### 4.2. Dataset Optimization

In this paper, the sEMG signal and its wavelet decomposition coefficients are used as feature sets to test the prediction models. The evaluation of prediction results based on LSTM is shown in Table 2. The overall prediction results are similar to those of SVR. The results show that the prediction effects of sEMG-based datasets of G1 and G7 gestures are better than that of wavelet decomposition. The MSE, RMSE, and MAE values of sEMG-based training set of G1 are 0.02, 0.06, and 0.07 lower than that of wavelet decomposition, respectively. And the values of G7 are 0.05, 0.04, and 0.14 lower than those of wavelet decomposition, respectively. On the contrary, the MSE, RMSE, and MAE values of wavelet decomposition dataset are 0.02, 0.06, and 0.08 which are lower compared to sEMG-based training set, respectively. The largest error of LSTM prediction model is sEMG-based dataset of G4, and its RMSE value is 0.1842. The least error is G1 wavelet decomposition dataset with the MSE value of 0.0002. Moreover, the MSE of G7 wavelet decomposition test set has an abnormal value, up to 6.2217.

**Figure 9:**
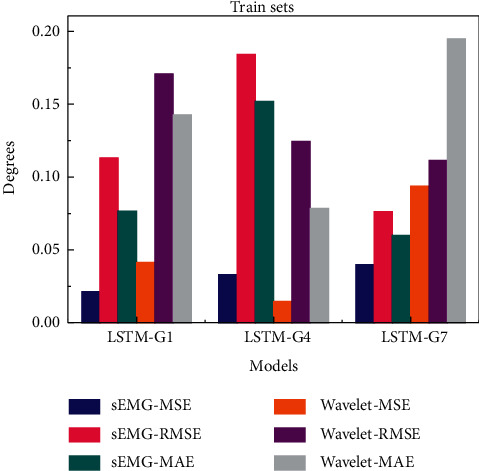
Comparison chart of MSE, RMSE, and MAE for LSTM prediction models based on sEMG and its wavelet decomposition datasets.

According to Figure 9, when the sEMG signals are used as datasets for gestures G1 and G7, the MSE, RMSE, and MAE values of LSTM prediction model are lower than those of wavelet decomposition coefficient datasets. The error values of G4 of LSTM prediction model are slightly higher than those of wavelet decomposition. Compared with the SVR model, the prediction and evaluation error of G1 and G7 based on wavelet decomposition dataset is increased, which is similar to or even higher than that of G4.

The evaluation of prediction results based on SVR is shown in Table 3. The overall prediction error of processed sEMG signals is less than that of wavelet decomposition coefficient. The MSE, RMSE, and MAE values of G1 based on wavelet decomposition datasets are 0.02, 0.8, and 0.06 which are higher than those of sEMG signals, respectively. The MSE, RMSE, and MAE values of G7 based on wavelet decomposition datasets are 0.05, 0.08, and 0.06 which are higher than those of sEMG signals, respectively. The MSE, RMSE, and MAE of G4 wavelet decomposition coefficient-based training set are better, which are 0.02, 0.06, and 0.06, higher than those of sEMG signals.

The biggest error of SVR prediction models appears in the sEMG-based dataset of G4 gesture, and the RMSE value is 0.1643. The least error is in the wavelet decomposition-based dataset of G1, and its MSE value is 0.0002, followed by wavelet decomposition-based dataset of G4 with MSE value of 0.0004. Both of them are evaluations of test sets. The smallest error of the training set is wavelet decomposition-based dataset of G4, and the MSE value is 0.0148.

**Figure 10:**
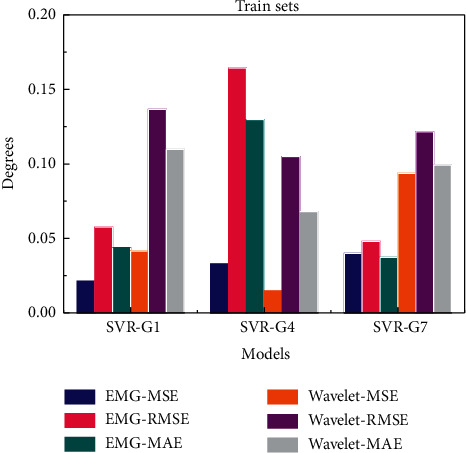
Comparison chart of MSE, RMSE, and MAE for SVR prediction models based on sEMG and wavelet decomposition datasets.

The MSE, RMSE, and MAE values of SVR prediction models in the training set based on sEMG signal and wavelet decomposition datasets are shown in Figure 10. Because of the outliers in the prediction of wavelet decomposition-based test set of G7, only the evaluation value of training set is compared.

The results show that the sEMG-based error of two-hand gesture G4 is the largest, and the RMSE and MAE values are 2-3 times higher than those of G1 and G7. However, the MSE, RMSE, and MAE of G4 of wavelet decomposed coefficient feature sets are lower than those of G1 and G7. It can be seen that the one-hand gesture of 4-channel signal can get better prediction results by using the processed sEMG signals as datasets. To obtain good prediction results, wavelet decomposition coefficients should be used to reflect the low-frequency characteristics of 7-channel signals.

### 4.3. Integrated Gesture Fatigue Predictions

The integrated gestures to be predicted can be divided according to two electronic equipment: smartphone and PC, as shown in Table 4. Browsing information on the two electronic equipment is one-handed 4-channel gesture, and its dataset is the most suitable for using sEMG signals. According to the situations of independent gestures, training models of G1, G2, and G3 are connected in series to make prediction. Series models of G7 and G8 are selected for PC-based browsing gesture prediction. Playing games and typing are both two-hand gestures on the two electronic equipment, which have 7-channel input signals. It is suitable to use wavelet decomposition coefficients of sEMG signals as datasets. Playing games and typing on smartphones use series models of G4, G5, and G6 to predict. PC-based games and typing use prediction training model of independent gesture G9.

The MSE, RMSE, and MAE evaluation values of training set are shown in Table 5, which are in a lower range. The trained series models are used to predict the fatigue degree of integrated gestures. To compare the accuracy, this paper uses the sEMG-based datasets for browsing information of one-handed gesture and the wavelet decomposition datasets for playing games and typing gestures and extracts 10000 groups of signals of each integrated gesture as feature sets for prediction.

The training results of the series model are ideal, and the evaluation errors are in a low range. The prediction error of playing games and typing gestures based on PC is the lowest, with an MSE value of 0.0032, followed by the MSE prediction error of games and typing based on smartphone of 0.0151. Because the prediction results of LSTM model are normalized sequences which cannot be compared directly. In this paper, the predictive fatigue values are obtained by inverse normalization calculation, as shown in Table 5. The results show that the fatigue of browsing information is the least, the fatigue of smartphone games is greater than that of typing, and the fatigue of PC typing is slightly lower than that of smartphone. The trend is consistent with the calculated results of energy spectrum.

**Figure 11:**
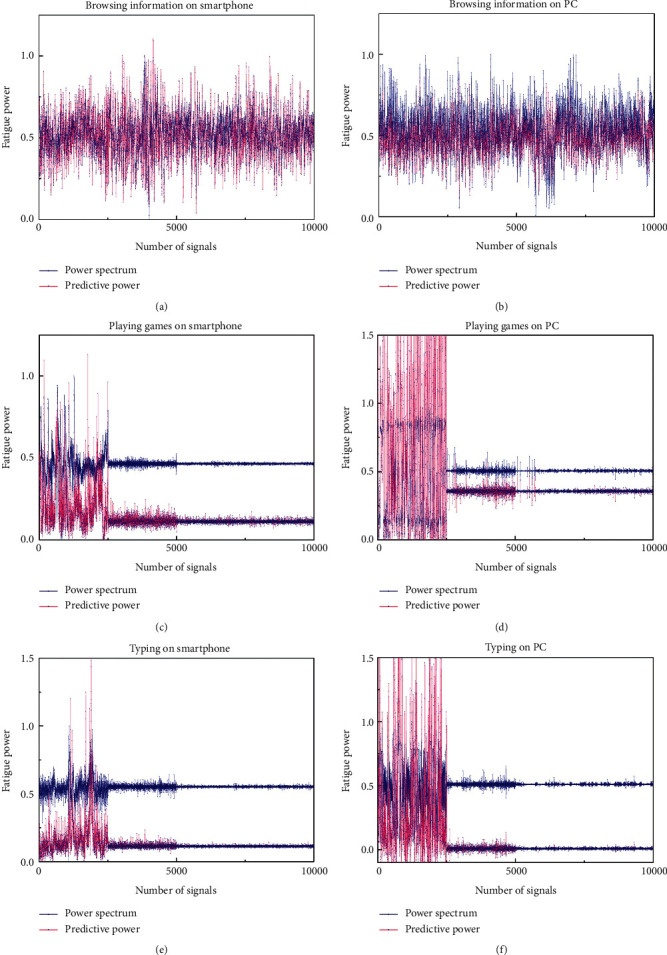
Fatigue comparisons between predicted results and power spectrums.

Figure 11 shows the comparisons between the prediction results and the power spectrums. For sEMG signals used as the feature data of browsing information gestures, those two results are consistent with each other, as shown in Figures 10(a) and 10(b). The trend of the two results is consistent in the wavelet decomposition coefficients-based gestures, but the predicted values of normalized distribution are lower than that of power spectrum. By adding the influence factors of independent gestures which compose the integrated gesture, the prediction model is validity. The predicted distribution power value is consistent with the distribution trend of spectrum, which proves the reliability of the models.

**Figure 12:**
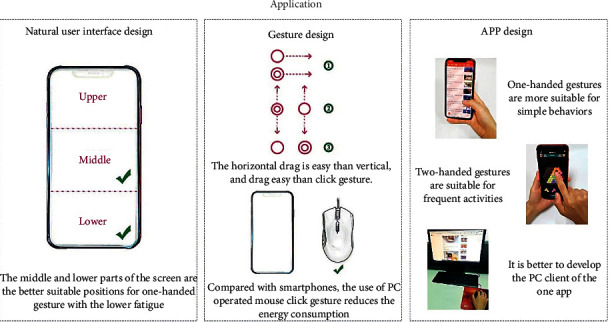
Application of the research results.

The fatigue degree of all independent and integrated gestures calculated by LSTM can provide reference for the design of natural user interface, gesture, and app, as shown in Figure 12.

### 4.4. Discussion

The BP neural network toolbox of MATLAB software is used for prediction training firstly. The normalized processed sEMG signals are used as the input datasets and the normalized power spectrum as the output. Nine hidden neurons with continuous weights and learning rate of 0.01 are used for training. The values of regression *R* represent the consistency between outputs and targets based on MSE. It is found that the training results are not very stable. During 20 tests, *R* of the best training set is 0.98 and that of the test set is 0.96. When the training results are poor, *R* of the training set is 0.71 and that of the test set is 0.63. There are eight times that the *R* of training sets is more than 0.9. The average *R* of the 20 training sets is 0.88, and that of test sets is 0.87 in 20 tests. When the number of neurons added or the epochs is increased to 50000, the *R* value is not significantly improved or stable, and the robustness is poor. Using Python-based Jupyter Notebook training, the training accuracy is 0.92.

An interesting phenomenon in the process of superparameters debugging during tests has been found. When the data are normalized to (0, 1), the accuracy of train sets based on MSE is 0.7782 and that of test sets is 0.7141. When normalized to (−1, 1), the accuracy of train sets is improved to 0.9887, and the accuracy of test sets is improved to 0.9769. This may be caused by the percentage of error function.

## 5. Conclusions

To better predict the fatigue problems of integrated gestures in complex environment, the following work has been done in this paper: First, BP neural network, RNN, and LSTM are trained, respectively. SVR is used as reference sample. MSE, RMSE, and MAE are used to evaluate. Second, the processed sEMG signals and wavelet decomposition coefficients are used as feature sets, and they are input into LSTM model and SVR for training.

Innovations: (1) For the fatigue analysis of comprehensive integrated gestures, the method of decomposing complex movements into independent gestures which are more convenient for monitoring is proposed for the first time and then integrated by neural network. (2) By comparing several neural network prediction models, it is found that LSTM is the most effective one for gesture fatigue prediction. (3) To further improve the accuracy, it is found that simple signals are suitable for EMG signals, and complex signals are suitable for using decomposed wavelet coefficients for further feature extraction as input sets.

Through experiments, it is found that the LSTM prediction model with appropriate datasets can improve the fatigue prediction ability of single spectrum calculation. The prediction model is more accurate and reliable by adding the influence factors of independent gestures which compose the integrated gesture. The predicted distribution power values are consistent with the distribution trend of spectrum, which proves the validity of the model. The research results can be applied to the reference of gesture design in complex human-computer interaction, and the reasonable gesture design can be determined by the fatigue degree of evaluation test. Quantitative analysis of gesture fatigue can avoid unreasonable gesture application, improve user interaction experience, and promote the realization of human-computer barrier-free interaction.

Neural network is the development trend. With the development of complex bionic neuron and the improvement of algorithm, neural network can be used in many fields. This study will also test more neural networks and optimization algorithms to improve the accuracy of the prediction models, such as CNN-LSTM integrated neural networks. In this study, we only collected the sEMG signals of five graduate students. In the future work, we should increase the number of subjects to obtain more comprehensive research data. The age and occupation of the subjects should also be expanded to obtain a wider sample reference.

## Figures and Tables

**Table 1:** Temperature and wildlife count in the three areas covered by the study.

sEMG		MSE	RMSE	MAE
BP-G1	Train	0.0196	0.3385	0.3127
	Test	0.0085	0.3421	0.3306

BP-G4	Train	0.0339	0.2218	0.1782
	Test	0.0243	0.1979	0.1617

BP-G7	Train	0.0395	0.2468	0.2032
	Test	0.0395	0.2327	0.1911

RNN-G1	Train	0.0214	0.1354	0.0928
	Test	0.0069	0.086	0.0579

RNN-G4	Train	0.033	0.1812	0.1487
	Test	0.0261	0.1612	0.1289

RNN-G7	Train	0.04	0.1169	0.1024
	Test	0.0376	0.1107	0.0984

LSTM-G1	Train	0.0214	0.1133	0.0768
	Test	0.0069	0.0696	0.0491

LSTM-G4	Train	0.033	0.1842	0.152
	Test	0.0261	0.166	0.1331

LSTM-G7	Train	0.04	0.0763	0.06
	Test	0.0376	0.0717	0.0569

SVR-G1	Train	0.0214	0.0576	0.0439
	Test	0.0069	0.04	0.0305

SVR-G4	Train	0.033	0.1643	0.1298
	Test	0.0261	0.1488	0.1162

SVR-G7	Train	0.04	0.0482	0.0373
	Test	0.0376	0.046	0.0361

**Table 2:** Comparison of MSE, RMSE, and MAE for LSTM prediction models based on sEMG and its wavelet decomposition datasets.

Prediction models		sEMG			Wavelet		
		MSE	RMSE	MAE	MSE	RMSE	MAE
LSTM-G1	Train	0.0214	0.1133	0.0768	0.0415	0.1709	0.1427
	Test	0.0069	0.0696	0.0491	0.0002	0.1047	0.1027

LSTM-G4	Train	0.033	0.1842	0.152	0.0148	0.1246	0.0785
	Test	0.0261	0.166	0.1331	0.0004	0.0313	0.0272

LSTM-G7	Train	0.04	0.0763	0.06	0.0938	0.1115	0.195
	Test	0.0376	0.0717	0.0569	6.2217	0.0213	0.1377

**Table 3:** Comparison of MSE, RMSE, and MAE for SVR prediction models based on sEMG signals and wavelet decomposition datasets.

Prediction models		sEMG			Wavelet		
		MSE	RMSE	MAE	MSE	RMSE	MAE
SVR-G1	Train	0.0214	0.0576	0.0439	0.0415	0.1367	0.1098
	Test	0.0069	0.04	0.0305	0.0002	0.0765	0.0735

SVR-G4	Train	0.033	0.1643	0.1298	0.0148	0.1049	0.068
	Test	0.0261	0.1488	0.1162	0.0004	0.035	0.0302

SVR-G7	Train	0.04	0.0482	0.0373	0.0938	0.1215	0.0993
	Test	0.0376	0.046	0.0361	6.2217	0.0696	0.0687

**Table 4:** Optimal configuration of integrated gesture fatigue predictions.

Integrated gestures	Smartphone			PC		
	Signal dimensions	Datasets	Training models	Signal dimensions	Datasets	Training models
Browsing	One-hand with 4C	sEMG signals	G1 + G2 + G3	One-hand with 4C	sEMG signals	G7 + G8
Playing games	Two-hand with 7C	WT coefficients	G4 + G5 + G6	Two-hand with 7C	WT coefficients	G9
Typing	Two-hand with 7C	WT coefficients	G4 + G5 + G6	Two-hand with 7C	WT coefficients	G9

**Table 5:** The prediction results of integrated gestures fatigue.

LSTM gestures	Smartphone				PC			
	MSE	RMSE	MAE	Prediction	MSE	RMSE	MAE	Prediction

Brower	0.0351	0.0662	0.0526	−1.61 × 10^16^	0.0372	0.0567	0.0425	−4.14 × 10^15^
Game	0.0151	0.1204	0.0886	−1.74 × 10^14^	0.0032	0.0559	0.0197	−4.43 × 10^15^
Typing	0.0151	0.1205	0.089	−9.70 × 10^12^	0.0032	0.0558	0.0185	−3.34 × 10^15^

## Data Availability

The main experimental data of processed sEMG signals and wavelet coefficients have been uploaded in Baidu cloud disk. Reviewers can download the information by accessing the address “https://pan.baidu.com/s/1AMkEryTBSlrZcU7r-mh5Ug” and using password “bnvf.”
